# Blocking of platelet glycoprotein receptor Ib reduces “thrombo-inflammation” in mice with acute ischemic stroke

**DOI:** 10.1186/s12974-017-0792-y

**Published:** 2017-01-21

**Authors:** Michael K. Schuhmann, Josua Guthmann, Guido Stoll, Bernhard Nieswandt, Peter Kraft, Christoph Kleinschnitz

**Affiliations:** 1 Department of Neurology, University Hospital Würzburg, Josef-Schneider-Straße 11, 97080 Würzburg, Germany; 2 Department of Neurology, University Hospital Essen, Hufelandstraße 55, 45147 Essen, Germany; 3 Rudolf Virchow Center, DFG Research Center for Experimental Biomedicine, University of Würzburg, Würzburg, Germany

**Keywords:** Ischemic stroke, Transient middle cerebral artery occlusion, Glycoprotein receptor Ib, Thrombo-inflammation

## Abstract

**Background:**

Ischemic stroke causes a strong inflammatory response that includes T cells, monocytes/macrophages, and neutrophils. Interaction of these immune cells with platelets and endothelial cells facilitates microvascular dysfunction and leads to secondary infarct growth. We recently showed that blocking of platelet glycoprotein (GP) receptor Ib improves stroke outcome without increasing the risk of intracerebral hemorrhage. Until now, it has been unclear whether GPIb only mediates thrombus formation or also contributes to the pathophysiology of local inflammation.

**Methods:**

Focal cerebral ischemia was induced in C57BL/6 mice by a 60-min transient middle cerebral artery occlusion (tMCAO). Animals were treated with antigen-binding fragments (Fab) against the platelet surface molecules GPIb (p0p/B Fab). Rat immunoglobulin G (IgG) Fab was used as control treatment. Stroke outcome, including infarct size and functional deficits as well as the local inflammatory response, was assessed on day 1 after tMCAO.

**Results:**

Blocking of GPIb reduced stroke size and improved functional outcome on day 1 after tMCAO without increasing the risk of intracerebral hemorrhage. As expected, disruption of GPIb-mediated pathways in platelets significantly reduced thrombus burden in the cerebral microvasculature. In addition, inhibition of GPIb limited the local inflammatory response in the ischemic brain as indicated by lower numbers of infiltrating T cells and macrophages and lower expression levels of inflammatory cytokines compared with rat IgG Fab-treated controls.

**Conclusion:**

In acute ischemic stroke, thrombus formation and inflammation are closely intertwined (“thrombo-inflammation”). Blocking of platelet GPIb can ameliorate thrombo-inflammation.

## Introduction

Ischemic stroke (IS) is a leading cause of death and invalidity, and its incidence is increasing [1]. The exact pathophysiologic mechanisms underlying IS are still not completely understood. While cerebral ischemia has, for many years, been considered to be a predominantly thrombotic disease, it is currently widely accepted that inflammatory processes also play an important role [2]. IS causes a strong inflammatory response, including transmigration of leukocyte subsets and expression of inflammatory cytokines [3]. We recently introduced the interplay between thrombotic and inflammatory mechanisms at the neurovascular unit as a pathophysiologic concept of thrombo-inflammation [4, 5]. A potential molecular target linking inflammation and thrombosis might be the platelet glycoprotein (GP) receptor Ib. Thrombotic pathways include the binding of GPIb to endothelial von Willebrand factor for the initial adhesion of platelets at vascular injury sites. With respect to inflammation, GPIb harbors a binding site for the integrin Mac-1 being expressed on neutrophils and monocytes. Therefore, GPIb has been shown to be involved in the recruitment of immune cells [6]. We have previously demonstrated that interfering with the early steps of platelet aggregation and activation via blocking platelet GPIb reduces infarct volume and improves stroke outcome in a mouse model of acute experimental stroke, without increasing the risk of intracerebral hemorrhage (ICH) [7]. Furthermore, targeting GPIb was also safe and effective in aged and comorbid mice with IS [8]. Therefore, blockade of GPIb may be a promising target for human stroke studies.

The aim of this study was to investigate whether GPIb blockade also has the potential to mitigate the stroke-associated inflammatory response.

## Materials and methods

### Animals

In this study, male C57BL/6 wild-type mice were used. The animals were aged 12–14 weeks. Animal experiments were approved by the legal state authorities (Regierung von Unterfranken) and performed according to the recommendations for research in experimental stroke studies [9], and the current ARRIVE (Animal Research: Reporting of In Vivo Experiments) guidelines (https://www.nc3rs.org.uk/arrive-guidelines).

### Animal treatment

Mice were given 100 μg p0p/B antigen-binding fragment (Fab) intravenously after a 60-min transient middle cerebral artery occlusion (tMCAO) at the time point of filament removal to inhibit GPIb. The control animals received 100-μg rat IgG Fab [8].

### tMCAO

Focal cerebral ischemia was induced by a 60-min-lasting tMCAO as described [7]. Twenty-four hours after tMCAO, the animals were sacrificed and the brains were cut in three 2-mm-thick coronal sections. The slices were stained for 20 min at 37 °C with 2% 2,3,5­triphenyltetrazolium chloride to visualize the infarctions [10]. Edema-corrected infarct volumes were calculated by planimetry (ImageJ software, National Institutes of Health) according to the following equation: *V*_indirect_(mm^3^) = *V*_infarct_ × (1 – (*V*_I_ – *V*_C_)/*V*_C_); (*V*_I_ – *V*_C_) represents the volume difference between the ischemic hemisphere and the control hemisphere and ((*V*_I_–*V*_C_)/*V*_C_) expresses this difference as a percentage of the control hemisphere. Global neurologic deficits were assessed according to the Bederson score [11]. The Grip test scores were used to monitor motor function and coordination [12]. Occurrence of ICH was macroscopically assessed on whole brains and again after coronal brain slices were cut before TTC staining. Researchers and operators were blinded to the experimental groups for all readout parameters.

### Real-time polymerase chain reaction (RT-PCR)

Tissue homogenization, RNA isolation, and real-time PCR were performed as described recently [5]. Relative gene expression levels of tumor necrosis factor-α (TNFα) (assay ID: Mm 00443258_m1, Applied Biosystems), interleukin (IL)1β (assay ID: Mm 00434228_m1, Applied Biosystems), and IL6 (assay ID: Mm 00446190_m1, Applied Biosystems) were analyzed with a fluorescent TaqMan technology. As an endogenous control Gapdh (TaqMan^®^ Predeveloped Assay Reagent for gene expression, part number: 4352339E, Applied Biosystems) was used. PCR was performed using StepOnePlus™ Real-Time PCR System (Applied Biosystem).

### Immunohistochemistry and thrombosis index

Histology and immunohistochemistry were performed according to standard procedures [13]. Cryoembedded coronal brain sections (2 mm) were cut into 10-μm-thick slices. Every tenth slice was used for evaluation. The following antibodies were used: polyclonal antibody anti-CD31 (ab9498, abcam), monoclonal antibody (mAb) anti-GPIX (generated by B. Nieswandt.), mAb anti-CD11b (MCA711, Serotec), and mAb anti-CD3 (MCA2690A488, BioRad). For quantification of occluded microvessels, brain slices were stained with hematoxylin and eosin. Afterwards, the numbers of occluded and opened vessels per hemisphere were counted to determine the percentage of occlusions as previously described [13]. All immunohistologic stainings were analyzed and acquired using a Nikon Eclipse 50i microscope.

### Statistical analysis

All results are given as mean ± standard error of the mean except for the Bederson score and the grip test, which are expressed as ordinal values. For statistical analysis, the GraphPad Prism 5.0 software package (GraphPad Software) was used. Data were tested for Gaussian distribution with the D’Agostino and Pearson omnibus normality test and then analyzed by unpaired, two-tailed Student’s *t* test. Scores addressing the functional outcome were compared using the Mann–Whitney *U* test. *P* < 0.05 was considered statistically significant.

## Results

**Fig. 1:**
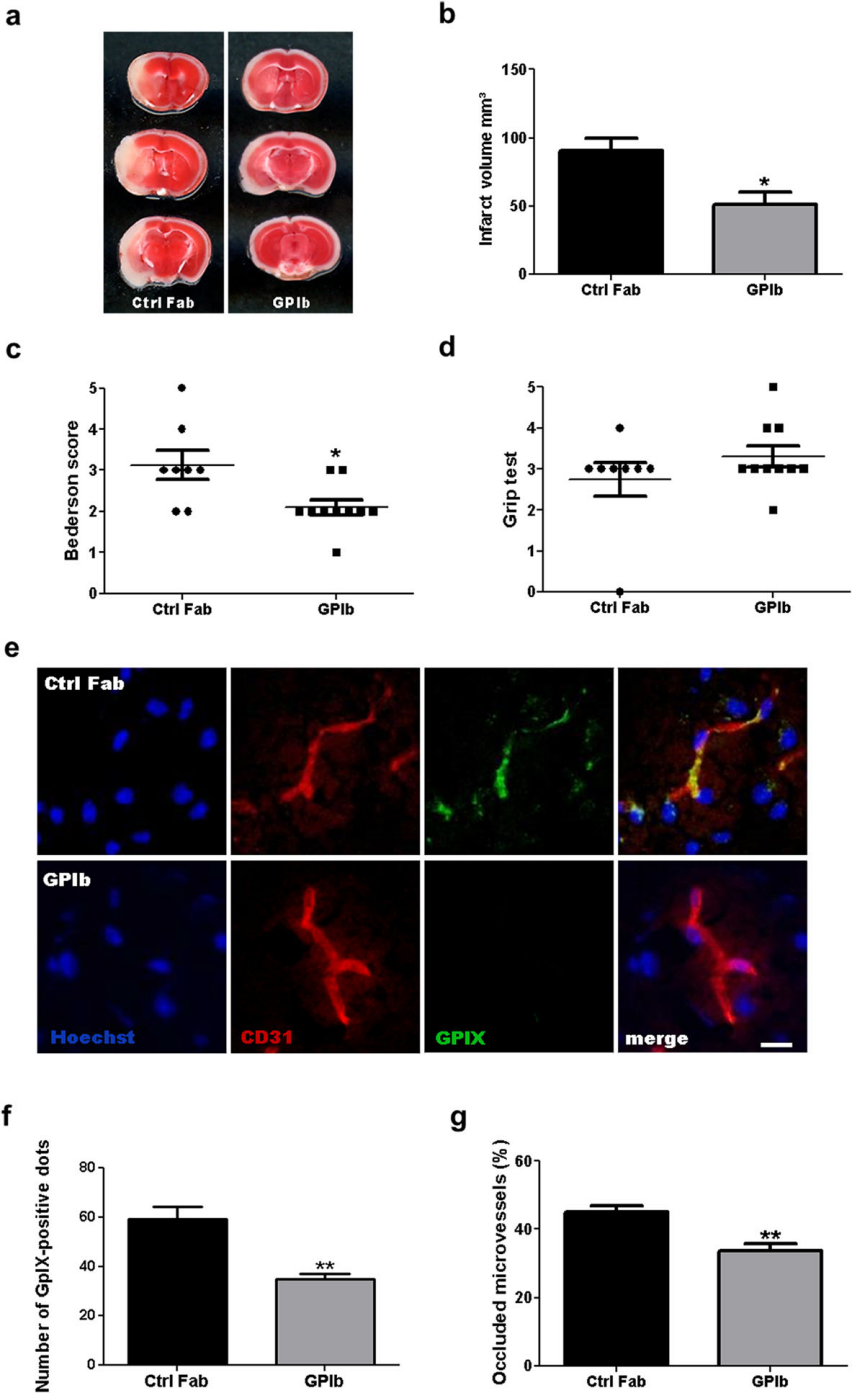
Infarct size, neurologic scoring, and brain microvascular thrombosis 24 h after stroke induction. **a** Representative TTC stainings of three corresponding coronal brain sections of mice treated with rat IgG Fab (Ctrl Fab) or p0p/B Fab (GPIb) 24 h after induction of tMCAO. Ischemic infarctions appear *white*, while vital tissue is stained *red*. **b** Infarct volumes as measured by planimetry (*n* = 8–10/group). **c** Bederson score and **d** Grip test score 24 h after tMCAO (*n* = 8–10/group). **e** Representative immunocytologic stainings of platelet aggregates within the vasculature from the ipsilateral hemisphere of mice, treated with rat IgG Fab (Ctrl Fab) or p0p/B Fab (GpIb) on day 1 after tMCAO. Hoechst depicts cell nuclei, CD31 stains endothelial cells, and GPIX represents platelet aggregates. *Scale bar*, 10 μm. **f** Quantification of ipsilesional glycoprotein IX (GPIX)-positive aggregates in GPIb-treated mice when compared with control mice. (*n* = 5 or 6/group). **g** Quantification of occluded ipsilesional vessels in hematoxylin–eosin (H&E**)**-stained brain sections on day 1 after tMCAO (*n* = 5–6/group). **b**, **f**, **g**, *P < 0.05, **P < 0.01, unpaired Student’s *t* test. **c**, **d**, *P < 0.05, Mann–Whitney *U* test

**Fig. 2:**
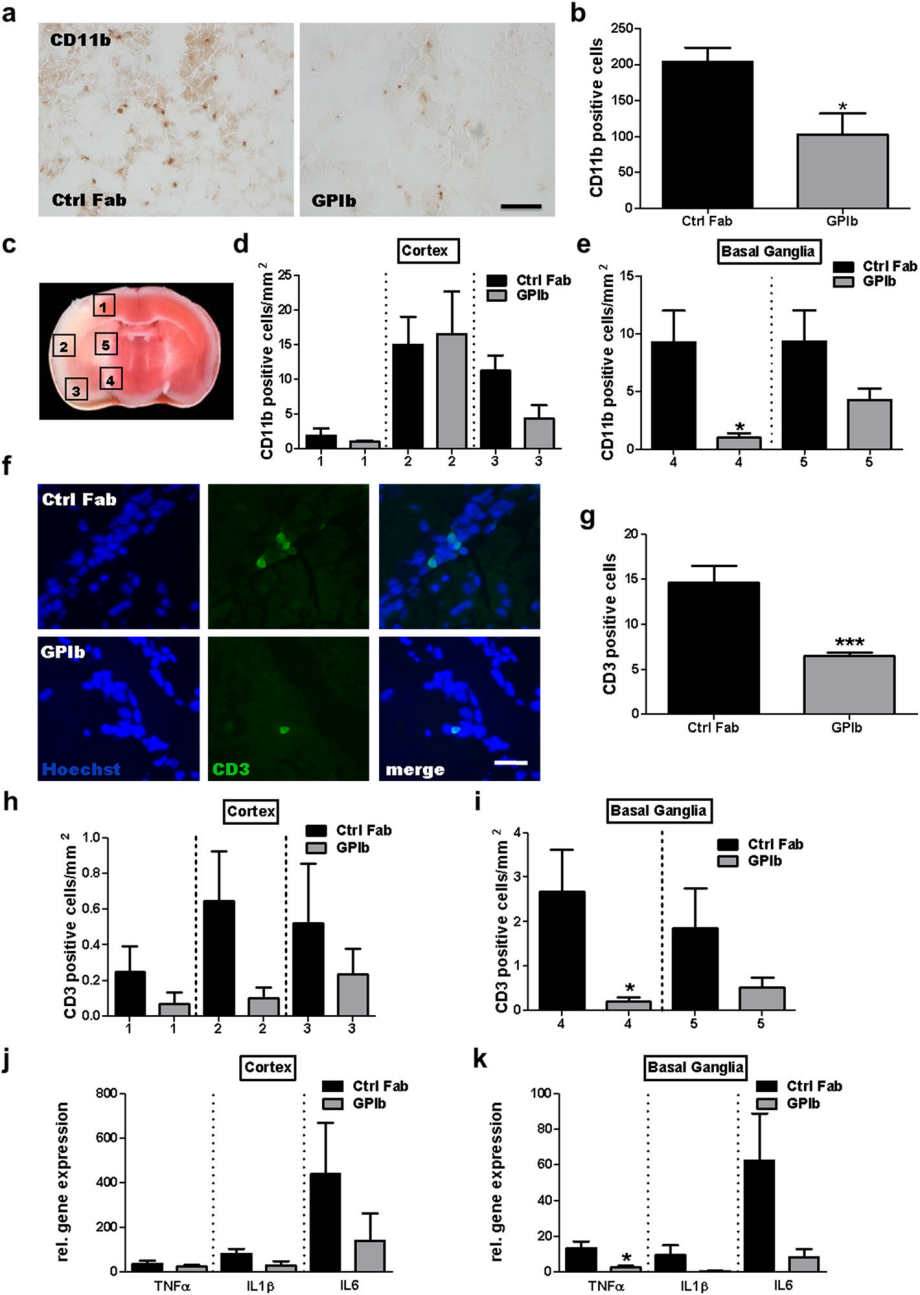
Quantification of immune cell accumulation and relative cytokine gene expression in the infarcted hemisphere at day 1 after stroke. **a** Representative CD11b-immunoreactivity 24 h after tMCAO of mice treated with rat IgG Fab (Ctrl Fab) or p0p/B Fab (GPIb). *Scale bar*, 100 μm. **c** Schematic view of the brain regions analyzed to quantify the density of immune cells per mm^2^. **b**, **d**, **e** Quantification of CD11b-positive cells per slice and CD11b-positive cells/mm^2^ in different cortical and basal ganglial regions in the ipsilateral hemisphere at day 1 (*n* = 5/group). **f** Representative immunocytologic stainings of brain-infiltrating CD3-positive T lymphocytes on day 1 after tMCAO of mice, treated with rat IgG Fab (Ctrl Fab) or p0p/B Fab (GPIb). *Scale bar*, 25 μm. **g**–**i** Quantification of CD3-positive cells per slice and CD3-positive cells/mm^2^ in different cortical and basal ganglial regions in the ipsilateral hemisphere at day 1 (*n* = 5/group). **j**, **k** Relative gene expression of tumor necrosis factor-α (TNFα), interleukin-1β (IL1β), and interleukin-6 (IL6) in the cortical and basal ganglia ischemic hemispheres of mice, treated with rat IgG Fab (Ctrl Fab) or p0p/B Fab (GPIb) (*n* = 6/group). *P < 0.05, ***P < 0.001, unpaired Student’s *t* test

Consistent with our previous results [7, 8], treatment with anti-GPIb 60 min after tMCAO significantly reduced stroke volumes (51.08 ± 9.52 mm^3^) compared with IgG Fab-treated controls (90.83 ± 9.07 mm^3^, *P* < 0.05) on day 1 (Fig. 1a, b). Importantly, reduced stroke size in the anti-GPIb-treated mice translated into better functional outcome as assessed by the Bederson score (values are the median with 25th and 75th percentiles, respectively, in brackets (IgG Fab, 3.0 (2.25, 3.75); GPIb, 2.0 (2.5, 2.25), *P* < 0.05) (Fig. 1c), but not in the Grip test (IgG Fab, 3.0 (3.0, 3.0); GPIb, 3.0 (3.0, 4.0), *P* > 0.05) (Fig. 1d)). Analogous to smaller infarct volumes, anti-GPIb treatment inhibited thrombus formation. The number of GPIX-positive platelet aggregates (number of GPIX-positive dots, IgG Fab 59.32 ± 5.16; GPIb, 35.07 ± 2.10, *P* < 0.01) (Fig. 1e, f) and ipsilesional occluded brain vessels (percentage of occluded vessels, IgG Fab 44.97 ± 1.9; GPIb: 33.62 ± 5.42, *P* < 0.01) (Fig. 1g) was significantly lower in anti-GPIb-treated animals compared with the control group. In the next step, we analyzed if the inhibition of platelet activation, and therefore thrombus formation, coincides with reduced inflammation. We demonstrated fewer ipsilesional activated microglia/macrophages (Fig. 2a–e, *P* < 0.05) and T cells (Fig. 2f–i, *P* < 0.05 and < 0.001) after treatment with anti-GPIb. Cytokine expression analysis revealed significantly less TNFα in the basal ganglia (relative gene expression, IgG Fab 13.33 ± 3.81; GPIb, 2.49 ± 0.89, *P* < 0.05) and a tendency towards reduced expression in the cortex as well as reduced expression of IL1β and IL6 in both regions (Fig. 2j, k).

## Discussion

In the present study, we identified reduced thrombo-inflammation to be associated with the stroke-protective properties of anti-GPIb treatment in mice. Importantly, in our current and previous studies, GPIb blockade reduced stroke volumes and significantly improved neurologic deficits in a clinically relevant setting, when injected 60 min after tMCAO [7, 8]. Of interest, stroke protection was not accompanied by ICH complications. The observation of an antithrombotic effect when targeting GPIb in an in vivo experimental stroke model is congruent with in vitro findings of platelet thrombus formation under flow [14]. GPIb has a key function in thrombotic processes, as it is essential for the initial adhesion of platelets to the vessel wall under high shear rates [15]. Furthermore, GPIb harbors a binding site for Mac-1. Mac-1 has been shown to be involved in the adhesion of leukocytes to platelets, and leukocyte-platelet complexes might promote inflammation [16]. Therefore, GPIb/Mac-1 interactions might provide a potential mechanistic link between thrombosis and inflammation [4, 6]. Our finding that blocking GPIb reduced the number of ipsilesional leukocytes after experimental stroke strongly supports the concept of thrombo-inflammation and is in accordance with a previous investigation that found GPIb to be involved in the recruitment of immune cells in a model of acute peritonitis [6]. Interestingly, besides reduced numbers of monocytes, the number of CD4-positive T cells invading the ipsilateral hemisphere was also significantly reduced in this study. Importantly, not only the overall number but also the density of infiltrating immune cells in brain areas that are infarcted in both treatment groups was reduced, arguing against an unspecific effect related to smaller infarct volumes.

Tumor necrosis factor, interleukin-1, and interleukin-6 are potent inflammatory cytokines that have been shown to modulate tissue injury in experimental stroke [17]. There is evidence that tumor necrosis factor and interleukin-1 are produced in the ischemic hemispheres of rodents by CD11b-positive cells [18–20]. This is in good agreement with our data revealing reduced TNFα and IL1β expression especially within the basal ganglia regions where blocking of platelet GPIb significantly reduced the number of CD11b-positive cells/mm^2^.

It is widely accepted that T cells have a detrimental role in the acute phase of IS [21, 22]. Brain ischemia rapidly activates the cerebral microvasculature [23]. Endothelial adhesion molecules become rapidly upregulated, and damaged vessels expose subendothelial matrix proteins to the bloodstream, thereby providing an interface for T cell–endothelial as well as platelet–endothelial and T cell–platelet interdependencies. Our mechanistic studies recently revealed that T cells promote stroke due to interactions with the activated endothelium. Thereby, T cells cause microvascular dysfunction and further increase thrombus formation during the early phase after tMCAO [5, 13, 24]. The present study shows, for the first time, that targeting platelets by blocking their early adhesion to vessel walls [25] impacts T cell driven inflammatory processes after stroke.

In summary, our study confirms that in acute IS, thrombus formation and inflammation are closely intertwined. Blocking of platelet GPIb can ameliorate thrombo-inflammation.

## Abbreviations

Fab: Antigen-binding fragment
GP: Glycoprotein
ICH: Intracerebral hemorrhage
IgG: Immunoglobulin G
IL: Interleukin
IS: Ischemic stroke
tMCAO: Transient middle cerebral artery occlusion
